# Synergistic cellulose-based nanocomposite packaging and cold plasma decontamination for extended saffron preservation

**DOI:** 10.1038/s41598-022-23284-9

**Published:** 2022-10-31

**Authors:** Maryam Amini, Milad Rasouli, Mahmood Ghoranneviss, Mahdi Momeni, Kostya Ken Ostrikov

**Affiliations:** 1 Plasma Physics Research Center, Science and Research Branch, Islamic Azad University, Tehran, Iran; 2 Department of Physics and Institute for Plasma Research, Kharazmi University, Tehran, Iran; 3 Faculty of Physics, Shahrood University of Technology, Semnan, Iran; 4 School of Chemistry and Physics and QUT Centre for Materials Science, Queensland University of Technology (QUT), Brisbane, Australia

**Keywords:** Plasma physics, Biotechnology, Nanobiotechnology

## Abstract

Sterilization of saffron packaging and maintaining the quality of saffron content are the main priorities in saffron preservation. Common modalities do not offer lasting saffron preservation and it is urgent to develop novel packaging approaches from renewable resources and prevent packaging waste. Here, simultaneous decontamination and quality maintenance of saffron is demonstrated, for the first time, through the synergistic application of nano-clay-loaded carboxymethyl cellulose (CMC)/polyvinyl alcohol (PVA) nanocomposites (CNCs) and cold plasmas (CP). Compared to the separate uses of CP and CMC/PVA/nano clay, our results confirm the synergies between CP and CMC/PVA/nano clay cause complete inactivation of *Escherichia coli* bacteria, while not significantly affecting the concentrations of the essential saffron components (safranal, crocin, and picrocrocin). Overall, the CP-treated CMC/PVA/nano clay fosters saffron preservation, through contamination removal and quality maintenance of the food product. The synergistic application of CP and CMC/PVA/nano clay thus represents a promising strategy for packaging, sterilization, and preservation of high-value food products.

## Introduction

The ever-increasing demand for the preservation of healthy food products over long periods necessitates longer food shelf-life, sterilization of equipment containing microorganisms, and sustainable food processing and packaging technologies. These technologies include high- and low-temperature treatments; adding chemical preservatives; and various packaging systems^1^. The conventional food preservation modalities have inadequate efficiency, cause food waste, and often present threats to consumers’ health^2^. Therefore, there is an urgent need to develop new strategies to overcome the current food product preservation challenges.

Saffron (*Crocus sativus* L*.*) is not only one of the leading multifunctional products in various food industries but also a major and arguably one of the most expensive spices in the world with diverse applications in food, cosmetics, and pharmaceutical fields. Apart from the high potential in the above-mentioned fields, the beneficial effect of saffron has been widely acknowledged as a multimodal therapeutic agent against diverse diseases ranging from depression, Alzheimer’s, Parkinson’s, cardiovascular diseases, atherosclerosis, diabetes, and female and male reproductive disorders to numerous cancers^3–5^. The most prominent producers of this spice are Iran, India, Greece, Italy, Morocco, and Spain. Saffron is well-known for its high levels of aroma, color, and flavor, which are heavily dependent on the presence of safranal, picrocrocin, and crocin. Saffron production requires a collection of distinct phases, ranging from harvesting, gathering, handling, drying, and packaging to storage^6^.

Unfortunately, the stigmata harvesting technology is not sufficiently developed yet, and the conventional, mostly manual, harvesting process causes high contamination rates with different microorganisms. Therefore, on one hand, saffron is prone to contamination by a wide range of environmental bacteria when gathered, processed, and sold in retail marketplaces because of its exposure to wastewater, dust, and animal excreta; on the other hand, by nature, saffron is exported and stored for a long time, which necessitates particular attention to “complete” sterilization^7,8^. Saffron packaging that is appropriate should preserve the biological, physical, and chemical quality of saffron, which can extend its shelf-life and lead to maintaining the safety and quality of saffron. In addition to the humidity, environmental physical damage, light, and oxygen, each package should address the issues related to microbial contamination^9,10^.

Conventional packages such as paper and paperboard, aluminum foil, glass, low-density polyethylene (LDPE), high-density polyethylene (HDPE), and modified atmosphere packaging cannot fully meet the above-mentioned requirements^11,12^. As a result, there is an immediate need to create a new preservation strategy for saffron in order to overcome the current obstacles and meet all of the requirements. Plastic is the most commonly used polymer material for saffron packaging due to its low cost, light weight, strength, stability, flexibility, impermeability, and lifespan. However, the non-biodegradable nature of plastic leads to the accumulation of huge quantities of post-consumer waste and significant environmental issues over time^8^. In addition, plastic containers are not ideal for preserving saffron since the plastic substance causes the spice to lose some of its qualities^13–15^. By substituting synthetic polymers with natural polymers, the environmental impact of synthetic polymers is reduced and the quality of food products, particularly saffron, is maintained^16^.

Carboxymethyl cellulose (CMC) is a cost-effective, biodegradable, non-toxic, and film-forming cellulose derivative that is widely employed in food packaging. Due to its strong water-absorbing ability, CMC is utilized for retaining moisture and enhancing the structural consistency of food products, where it is typically blended with other stabilizers^17,18^. Despite the superior properties, further increasing the physio-chemical properties, such as solubility, and mechanical properties of the CMC constructs involves blending with other polymers and the addition of numerous natural fillers. However, natural biopolymer-based films and their derivatives have poor mechanical characteristics and are hygroscopic^19^. By combining two or more biopolymers with a certain amount of nanofillers, these qualities can be improved. Additionally, even though CMC is one of the best biopolymers for making films, the development of bio-composite films still needs more innovations before they can be sold commercially^17–20^.

Polyvinyl alcohol (PVA) is a biocompatible, hydroxyl-rich, semi-crystalline, and non-toxic polymer that is water-soluble. PVA’s low biodegradation rate and high moisture absorption are the most prevalent characteristics that support its use in food packaging^21,22^. PVA contains hydroxyl groups, which cause intermolecular and intramolecular hydrogen bonding. Hydrogen bonding creates a strong contact between CMC and PVA. Both polymers are water-soluble, which leads to the formation of a homogenous solution. The consequent compounds created by combining CMC biopolymer and PVA synthetic polymer exhibit superior mechanical characteristics and chemical stability and permit the setup of larger nanocomposites^19,23^.

Even though different antimicrobial compounds, such as essential oils, could be used to give packaging films antimicrobial properties, these compounds also change other properties of the film, such as its thermal, barrier, and mechanical properties^24^. The problems associated with CMC/PVA films concerning shortcomings in antimicrobial properties are to be relieved via the addition of a variety of nanomaterials, such as nanoclay^25^. To overcome this phenomenon, nanoclays have been incorporated into bio-based and synthetic polymers to create nanocomposites with superior antibacterial, antioxidant, barrier, thermal, and mechanical properties^26^. In particular, nanocomposite films containing active nanoclay are highly antibacterial, prevent lipid oxidation against foodborne microorganisms, and can preserve food product quality and extend their shelf life. When nanoclays are suitably disseminated in a polymer matrix, they become highly exfoliated. The nanoclays’ exfoliation contributes to their enhanced interaction with polymer chains^26,27^. Thus, the enhanced contact contributes to the increased mechanical strength of the nanocomposite films. The nanoclay in the polymer matrix also creates a winding path that stops gas and water molecules from getting through and improves the barrier properties^26–28^.

Montmorillonite (MMT) consists of two tetrahedral silica sheets that are bonded to the edge of an octahedral Al(OH)_3_ sheet and is frequently utilized as a nanofiller in films and coatings for food packaging^26,29^. The smectite clay mineral is an aluminosilicate with a high surface-to-volume ratio and cation-exchange characteristics. These characteristics give excellent miscibility with cationic polymers, water-dispersibility, and active compound-carrying capacity. The integration of MMT into biopolymers results in substantial enhancements to their antibacterial, water vapor permeability (WVP), light/UV blocking, and mechanical properties. Substantial growth inhibition of *E. coli* has been shown with MMT-loaded starch/carvacrol and CH/cinnamaldehyde films displaying high antibacterial efficacy^30,31^. Therefore, MMT-loaded CMC/PVA nanocomposite films were used for saffron packaging in this study. The incorporation of polymer/nanoparticle films helps extend the food products’ shelf-life by means of their unique features. However, due to the various factors in saffron packaging, especially the dry nature of saffron, the efficacy of antibacterial nanomaterials is limited^32,33^. Thus, nanocomposites (NCs) should be subjected to other post-packaging sterilization methods such as ionizing radiation.

Among the irradiation-based treatments that are utilized to improve the saffron microbiological quality, compared to others, ionizing radiation such as microwave, electron beam and gamma radiation treatments are promising, but owing to the edge overheating, non-heterogeneous heating, soggy texture, and browning, they are also destructive to the essential saffron content^6,34^.

Over the last decade, cold plasma technology has received increasing attention due to the mild dose and chemical and physical environment that it creates for a range of industrial and medical applications^35,36^. Cold plasma (CP) is well-known for its adjustable level of reactive oxygen and nitrogen species, UV radiation, and reliance on electromagnetic (EM) fields^37^. Food preservation^38^ along with diverse applications such as plasma medicine^39,40^, CO_2_ conversion^41^, water treatment^42,43^, textile^44^, etc. are some application areas of cold plasmas distinguishing the plasma technology from other technologies. Replacing chemical decontamination techniques, steam sterilization, irradiation, and high hydrostatic pressure as traditional antimicrobial modalities with cold plasmas not only leads to high safety and efficacy but also is associated with reduced changes in food product quality^45^. Although previous studies emphasize the antimicrobial performance of cold plasma on walnuts and black pepper^46,47^, saffron has not been examined yet.

Accordingly, here we integrate nanocomposites and CP treatment modalities to develop novel contamination-free and high shelf-life extended packaging for saffron preservation. Hence, based on nano clay, a combination of carboxymethyl cellulose (CMC) and polyvinyl alcohol (PVA) as matrices were utilized for the preparation of CNCs film. Also, the effects of CP under different exposure times and voltages are examined on saffron content and contamination. The antimicrobial activity of CNCs film and CP treatment separately and jointly was evaluated for *E. coli* pathogen inactivation. Furthermore, crocin, safranal, and picrocrocin concentrations are studied during the storage period through the above-mentioned methods. Our findings reveal that the presence of both CNCs and CP in the saffron preservation process can potentially substantially improve the decontamination and quality maintenance performance of saffron packaging.

## Materials and methods

### Film preparation

**Figure 1:**
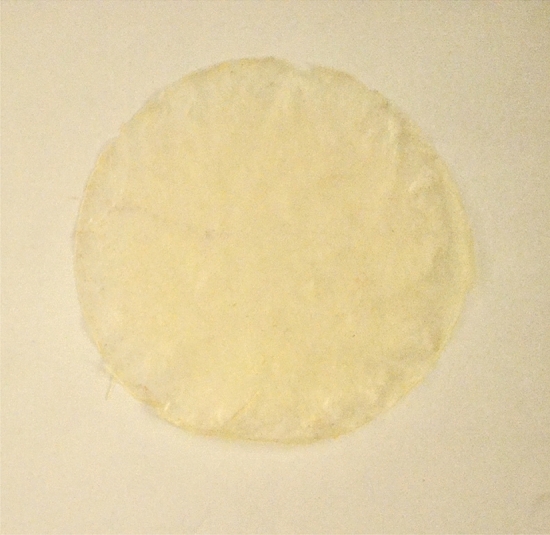
The cellulose nanocomposite film containing nano clay is used for saffron packaging.

CMC/PVA/nanoclay nanocomposites (Fig. 1), with a little modification, were prepared according to the method developed earlier^48^. Briefly, 3.5 g of carboxymethyl cellulose (CMC; average molecular weight of 41,000; practical grade; USK Kimya, Turkey) was dissolved in 200 mL of distilled water and heated for 45 min at 90 °C. Then, 10% w/v polyvinyl alcohol (PVA, Tetrachem agency, Iran) was added and heated at 90 °C for 40 min. Next, 3% of MMT (Cloisite Na + , Southern Clay, USA), 30 B (w/w CMC) was dispersed in 100 mL of distilled water, followed by 10 min of ultrasonication in a water bath. After mixing the solution containing CMC and PVA with MMT, the solution was heated for 15 min at 70 °C with stirring. 1.4 mL of glycerol (40 mL per 100 g of CMC) was mixed into the solution. The mixture was stirred for 20 min at 65 °C. After cooling the solution to ambient temperature, film-forming solutions are poured into a plate (glass jar) with a soft, uniform surface in order to control film thickness. The film was dried at 60 °C for 15 h and peeled off the surface to be utilized in various tests.

### Plasma device and treatment procedure

As previously described^45^, the plasma jet consisted of a 12 kHz pulsed DC high voltage power supply, power electrode, Pyrex tube, and nozzle. The powered electrode was wrapped around the nozzle. For evaluating the effect of the plasma jet on saffron, the feeding gases were 99.99% pure argon (Ar) with a 1-L/min gas flow rate. The plasma exposure time varied from 0 to 12 min. Besides, due to the previously identified harmful effect of plasma irradiation at 8 kV on saffron content^45^, in this study, we adjusted voltage to 5, 6, and 7 kV. The sample was put on Petri dishes and set in contact with CP. The distance between the nozzle and the sample was kept constant at 1 cm during the experiments.

### Microbial contamination

The antibacterial effects of CP and CNCs against *E. coli* gram-negative bacteria under the above-mentioned conditions were examined until 90 days of storage. Briefly, suspension of *E. coli* (ATCC 10,536) was prepared according to 0.5 McFarland turbidity. The Nutrient Agar medium was used for plating and enumeration of the *E. coli* colonies. 40 g saffron powder was inoculated with *E. coli* for six examined groups using a sterile glass sprayer and homogenized to yield 10^6^ live cells per one gram of sample. The spiked sample was divided into 80 parts and packaged into 80 packets of 0.50 g saffron using the composite films. Based on cultural characteristics, *E. coli* is distinguished from other bacterial species. On nutrient agar, *E. coli* colonies are large, thick, greyish-white, moist, smooth, opaque, or translucent discs. At each proposed time point, samples were taken and they underwent extensive analysis for isolation and enumeration of the *E. coli* bacteria from other bacteria, considering the cultural characteristics of *E. coli* by the CFU method.

### X-ray diffraction (XRD)

As a non-destructive technique, X-ray Diffraction (XRD) elucidates the phase purity, crystalline structure, and other structural parameters of materials^49^. Here, information about the role of CP irradiation and clay particles inside the polymer matrix was determined by means of an X-ray diffractometer (Model Xpert-Philips, Pw 3040/60, MI, USA) at four modes CMC/PVA, CMC/PVA/nano clay, CMC/PVA-CP treated, and CMC/PVA/nano clay-CP treated.

### Sample preparation and treatment

**Figure 2:**
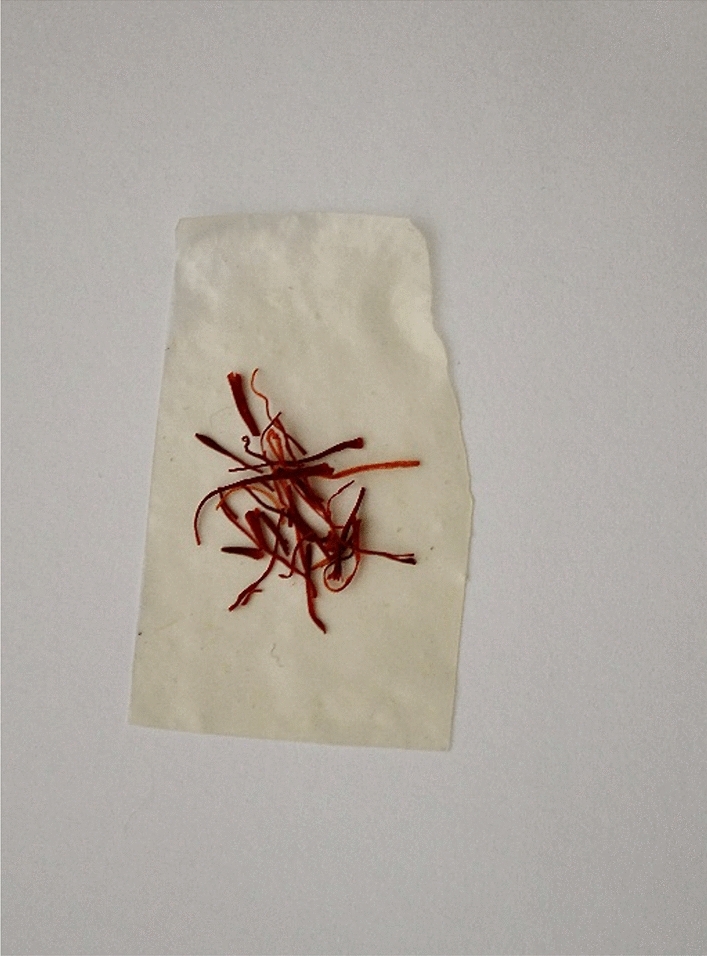
Packaging of the saffron with the nanocomposite CNC film.

60 samples of inoculated saffron (Qaen city, Khorasan, Iran) were treated by CP between 0 and 12 min exposure times at 5, 6, and 7 kV voltages. After the treatments, along with the number of *E. coli* bacteria, the concentrations of safranal, crocin, and picrocrocin were evaluated. Next, the treated and untreated samples were stored at room temperature for 30, 60, and 90 days and the changes of safranal, crocin, and picrocrocin of saffron during the storage period were checked. On the other hand, 10 inoculated samples were packed with CNCs (Fig. 2.), and the number of *E. coli*, accompanying the amounts of safranal, crocin, and picrocrocin of saffron was studied after 30, 60, and 90 days of storage.

### Synergy of CP and CNCs

**Figure 3:**
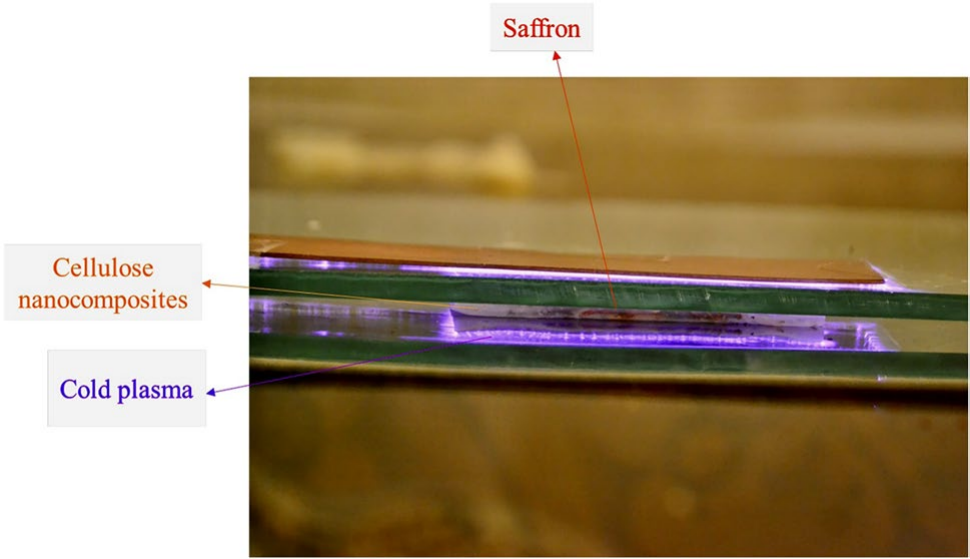
Plasma treatment of saffron within the nanocomposite CNCs packaging.

Owing to the eliminating a high percentage of *E. coli* cells without any adverse effect on the safranal, crocin, and picrocrocin content of saffron, 5 min exposure time at 6 kV voltage or 3 min exposure time at 7 kV voltage were selected as the CP treatment states for evaluating the synergistic effects of CP treatment and CNCs film. In the next step, saffron was packed by CNCs, set in contact with the mentioned CP irradiation condition, and after that was stored for 30, 60, and 90 days. Then, the amount of *E. coli*, safranal, crocin, and picrocrocin in the saffron was checked after the storage period. The experimental procedure is shown in Fig. 3.

### Determination of safranal, crocin, and picrocrocin

The main components of saffron, crocin, picrocrocin, and safranal have been recognized by international agencies that should not be affected by the packaging^50^. A Milton Roy model 21D UV/Vis spectrophotometer was used for monitoring the ultraviolet–visible (UV–Vis) spectra. 500 mg saffron was put into a 1 L volumetric flask and 900 ml distilled water was added. The mixture was stirred for 20 min. After adding 1 ml of the mixture to 1 L of distilled water, the solution was transferred into the quartz cuvette. The absorbance measurements were carried out at 257, 330, and 440 nm for picrocrocin, safranal, and crocin, respectively.

The calculation of the percentage of each component was done as follows:

$$X\left( \% \right) = 100\left( {A/M} \right)$$

X: the percentage of safranal, picrocrocin, and crocin A: the specific absorbance which has been reported by spectrophotometer at the maximum concentration M: the weight of the saffron sample (mg).

### Moisture content

The saffron samples were put into an electric oven at 100–105 °C for 16 h and the moisture of saffron was measured using the below equation:

$${\text{Moisture}}\;{\text{content}}\left( \% \right) = \left[ {\left( {{\text{W}}_{0} - {\text{W}}_{{\text{t}}} } \right)/{\text{W}}_{0} } \right] \times 100$$

W_0 _= Sample initial mass (g) W_t _= Sample final mass (g).

## Results and discussion

The saffron harvesting and post-harvesting processes play a critical role in its safety and quality. The saffron production process from cultivation to harvesting is done by hand and is susceptible to microbial contamination. Saffron preservation modalities should possess several features, ranging from decontamination to maintaining the quality of saffron^9,10^. Here, we combined CNCs and CP technology, to achieve the most suitable saffron packaging approach.

### X-ray diffraction analysis

**Figure 4:**
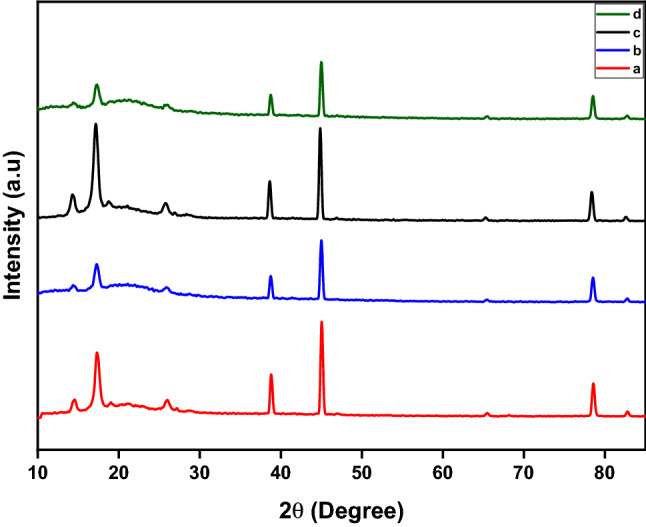
X‑ray Diffraction (XRD) for various packaging was used in this study. (**a**): XRD analysis of the package without nano clay and any treatment, (**b**): XRD analysis of the package containing nano clay and without any treatment, (**c**): XRD analysis of the package without nano clay and after the cold plasma treatment, (**d**): XRD analysis of the package containing nano clay and after the cold plasma treatment.

For studying the materials’ crystallographic structure, X-ray diffraction (XRD) analysis is used as a standard and non-destructive method^51^. The XRD spectra of CMC/PVA, CMC/PVA/nano clay, CMC/PVA-CP treated, and CMC/PVA/nano clay-CP treated are shown in Fig. 4a–d, respectively. According to the XRD patterns of CMC/PVA, the maximum diffraction peaks existing at 2Θ = 19°, 39°, 45°, and 79° are related to the XRD patterns of CMC/PVA. Pure PVA exhibits two distinct diffraction peaks at 19.9° and 34.7°, which correspond to an orthorhombic lattice (110) reflection, indicating that it is semi-crystalline. Three diffraction peaks were also detected at 2θ, 20.7°, 34.4°, and 44.7° for the pure CMC^52,53^. Due to the presence of CMC, the observed maximum intensity diffraction peak at about 19° is slightly higher than the main peak of pure PVA. The recorded sharp reflection at 19° of the CMC/PVA film is attributed to the (110) lattice planes of PVA and the semi-crystalline nature of CMC and reveals a significant interaction between CMC and PVA^52,53^. Additionally, the following strong peak of the CMC/PVA film was observed at almost 45°, which was slightly higher than the principal peak of pure CMC, probably attributable to the presence of PVA. Owing to the presence of both crystalline and amorphous regions, the two polymers are often semi-crystalline materials^52–55^. It can be assumed that CMC and PVA have undergone complexation. Destruction of the crystal structure of PVA elongates the molecular chain of PVA and increases its interaction with the molecular chain of CMC, hence facilitating the formation of intermolecular hydrogen bonds between the OH and COOH groups of CMC and the OH group of PVA. Indeed, when the hydroxyl group of PVA interacts with the carboxylate anion of CMC, ion-dipolar complexes are formed. These complexes can make the polymer chain less stiff and, as a result, less crystalline^56–59^.

Comparing the XRD patterns of CMC/PVA and CMC/PVA that had been treated with cold plasma (Fig. 4a,c, and d), the strength of the diffraction peak at 19° and 45° increased, but no new peaks appeared. This shows that the cold plasma did not change the type of crystal, but rather made the CMC/PVA more crystallized. The crystalline character of the surface of the plasma-treated CMC/PVA can be described as follows: First, the plasma treatment eliminated the low-molecular-weight fragments from the amorphous regions, leaving only the crystallized region. Second, plasma-charged particles generate extra polar groups on the surface of the CMC/PVA nanocomposite films, resulting in cross-linking with another atom^60,61^. Intramolecular forces of attraction between polar groups and polymers can benefit the polymer in crystallization by producing a linear chain on the surface. These polar (hydroxyl, carbonyl, and carboxyl) functional groups, which comprise the backbone of PVA and CMC, are strongly attracted to one another and create hydrogen bonds. This strong bond maintains the crystal firmly in place^62–64^.

When nanoclay is present, the intensity of the diffraction peaks diminishes, indicating a decrease in crystallinity, an increase in amorphousness of the CMC/PVA film polymers, and greater integration of MMT into the CMC/PVA polymer film (Fig. 4a,b, and d). A decrease in the film’s crystallinity may also indicate the formation of hydrogen bonds between the molecules of CMC and PVA. Peak intensities may have dropped or risen as a result of hydrogen bond formation, overlapping diffraction peaks, crosslinking, and molecular mixing. Due to their flexible backbone, amorphous polymers can have a high ionic conductivity and diffusion rate^65–68^.

The absence of clay peaks in the XRD patterns of CMC/PVA hybrids containing nanoclay suggested that the nanoclay diffused homogeneously into the polymer matrix, resulting in exfoliated nanocomposites^65,68,69^. It may be a result of the strong polar interactions between the hydroxyl groups of biopolymer chains, glycerol, and silicate layers. Furthermore, if there is too much space between the layers of nanoclay, the peaks on an X-ray diffractogram will disappear. This means that all of the silicate layers in the polymer matrix have been exfoliated^69–71^. On the other hand, among several MMT-based nanoclays, i.e., Na^+^, 15A, 20A, 93A, and 30B, it has been established that, with the exception of the 30B nanocomposite, none of the other nanoclays combinations accomplished exfoliation when combined with Polylactic acid^67^. Due to the strong interactions between the hydroxyl and carboxyl groups of nanoparticles and the hydroxyl and carboxyl groups of CMC, polymer chains do pass through the layers of nanoparticles and spread out among them. It is also hypothesized that the CMC or PVA polymer chains, or both, entered the silicate layers to form an exfoliated structure due to the driving force resulting from the strong hydrogen bonds between the CMC and PVA and the oxygen groups of the silicates^30,52,56,66,67,69,70^. Transmission electron microscopy has also demonstrated that the bulk of the clay layers was exfoliated and equally disseminated in CMC/PVA^68^.

### Moisture content (MC)

Crocin has a carotenoid structure, and the carotenoid stability is affected by light, oxygen, moisture content, metals, and other pro-oxidants, the presence of antioxidants, free radical inhibitors, and the composition and physical structure of the sample^72,73^. During the storage, the saffron-packed moisture was the same as with the plastic bag and CNCs package. Moreover, all samples were kept under the same environmental conditions and any additives such as metals, pro-oxidants, and antioxidants were not added to the samples. Therefore, light is the only remaining parameter that causes the different amounts of crocin during storage and affects the carotenoid.

### Optical properties

**Figure 5:**
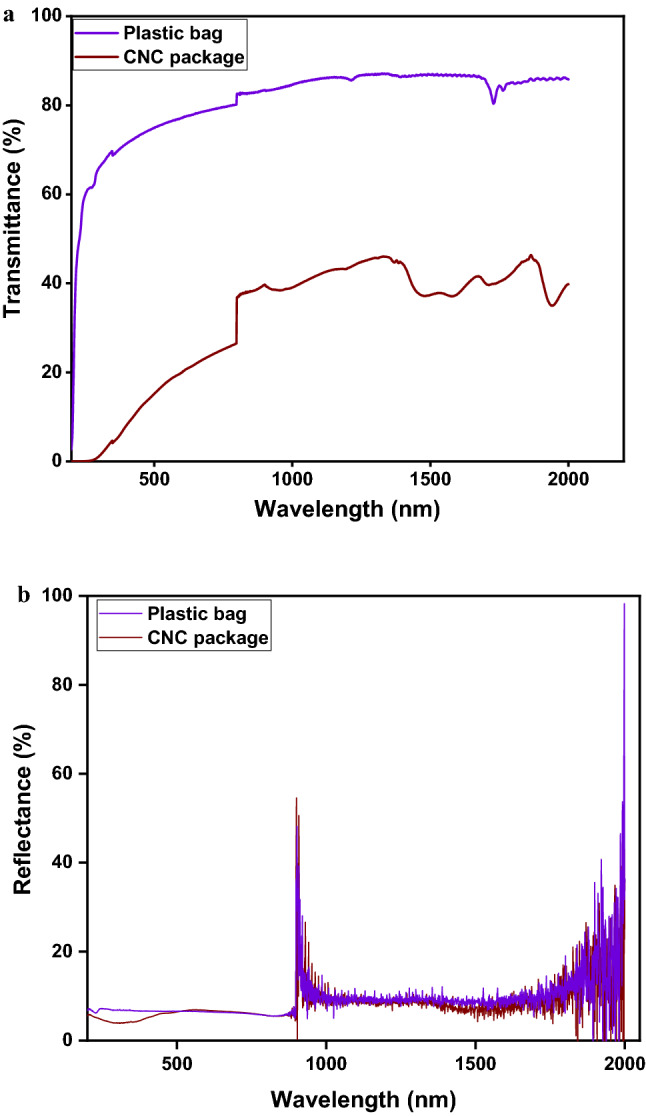
Light transmittance (**a**) and reflectance (**b**) spectra of plastic bag and CNCs package.

The optical transmittance and reflectance patterns of the CNCs and plastic packages were shown in (Fig. 5). The plastic bag had a high transmittance in the range of 0–2000 nm, indicating that it is highly transparent to UV and visible light, as is clear from its visual appearance. The CMC/PVA/nano clay film prevented light from passing through the CNF film with a matt surface finish. The decrease in light transmittance of the films was likely attributable to the nanoclays’ ability to scatter and reflect light, obstructing light transmission^74,75^. A comparison of the results in Table 2 and Fig. 5b shows that the amount of crocin is directly related to light transition. The amount of crocin is higher after enclosing the saffron by the CNCs packaging, which has a lower light transmittance. This result confirms that the light transmission property of the nanocomposite package is the most important parameter in saffron packaging.

### Synergistic effect of nanocomposite packaging and plasma on saffron decontamination

Regarding the inactivation efficacy of CP-CNCs against microbial contamination, our results demonstrate that the number of viable *E. coli* cells in the samples reduced to 4.4 and 4.0 (log CFU/gr) in the CP-treated CNCs compared to 6.0 (log CFU/gr) for control plastic bag and CNC groups before storage, respectively. After 60 days of storage, the number of *E. coli* cells for the plastic bag, CP-CNC-C (5 min exposure time at 6 kV voltage) and CP-CNC-D (3 min exposure time at 7 kV) changed to 7.7, 3.3, and 1.9 (log CFU/gr) compared to their controls, respectively (Table 1). Thus, not only did the CP-CNCs show good antibacterial performance compared to the plastic bag in pre-storage conditions, but they also significantly reduced bacterial contamination after 60 days compared to its control (*P* < 0.05). Interestingly, the CP-CNC-D completely eliminates *E. coli* cells and provides a contamination-free environment for saffron packaging over 90 days of storage.

**Table 1:** The effect of the packaging and the cold plasma treatment on the number of *E. coli* cells (Log CFU/gr).

Treatment	Packaging	Cold plasma					
		Voltage (kV)	Time (min)	Storage(days)			
				0	30	60	90
A	Plastic bag	–	–	6.0 ± 0.45^a^	6.9 ± 0.14^b^	7.7 ± 0.23^c^	8.4 ± 0.19^d^
B	CNC			6.0 ± 0.45^a^	5.1 ± 0.12^b^	4.0 ± 0.02^c^	3.4 ± 0.18^d^
C	CNC	6	5	4.4 ± 0.41^a^	3.9 ± 0.41^b^	3.3 ± 0.0^c^	3.00 ± 0.0^c^
D	CNC	7	3	4.0 ± 0.23^a^	3.0 ± 0.35^b^	1.9 ± 0.0^c^	0.0 ± 0.0^d^

A: The Saffron is packed in a plastic bag without any treatment and stored for 30, 60, and 90 days (control). B: The Saffron is packed by CNC package without any treatment and stored for 30, 60, and 90 days. C: The CNC packed Saffron is treated by cold plasma for 5 min at 6 kV and stored for 30, 60, and 90 days. D: The CNC packed Saffron is treated by cold plasma for 3 min at 7 kV and stored for 30, 60, and 90 days. The number of *E. coli* cells was enumerated. abcd means in the same row followed by different superscript letters are significantly different (*P* < 0.05). Data represent the mean ± standard deviation of each sample (n = 3).

Cellulose-based packaging can act as a carrier of antibacterial agents^18^. In addition, CP treatment can increase surface adhesion of CMC films depending on the exposure time, where the coating of antimicrobial compounds significantly improves after plasma processing^62^. For instance, CP treatment is accompanied by significant inhibition zones for *E. coli*, *Pseudomonas aeruginosa*, *Staphylococcus aureus*, *Salmonella typhimurium*, and *Bacillus cereus* when the plasma is utilized as a complementary agent to improve antimicrobial properties of Zataria multiflora essential oil (ZEO)-loaded CMC/PP bilayer film^63^. Therefore, on one hand, plasma exposure enhances the absorption of nano clay into the CMC/PVA film followed by its subsequent release, while the structural properties of CNCs are modified. On the other hand, CNCs film might carry plasma-derived antibacterial agents. In this way, the synergy of CP treatment and CNCs leads to the complete inactivation of *E. coli* bacteria. The mechanism of plasma-assisted inactivation of *E. coli* bacteria is related to the cell envelope damage-induced leakage owing to plasma-generated ROS/RNS, especially H_2_O_2_, NO_X_, and ozone reactive species^76^.

The obtained results about the replication and survival of *E. coli* in 90 and 180 days^77^ of storage may be due to a combination of different factors. First, *E. coli*’s survival and growth can potentially be affected by the fluctuating temperature circumstances utilized in this investigation^78^. Second, the ability to survive and even flourish in *E. coli* in low carbon content environments is due to the production of RpoS (subunit of RNA polymerase)^79^. Third, the absence of predatory, antagonistic, or competing species has been accompanied by the survival of *E. coli* bacteria^79^. Fourth, exhibiting higher levels of transcription for stress defense genes compared to commensal bacteria in *E. coli* bacteria under carbon-limited growth circumstances^80^. Fifth, as a result of plasma treatment, the C–C and C–H bonds in the polymer chain are broken, giving birth to carbon radicals^62^. Sixth, the complex interplay amongst CMC, PVA, and nanoclays has led to carbon resources. All of these points allow the survival of the pathogen for long periods of time. Considering these explanations, we need to rethink what we know about how E. coli survive in an open environment, both in terms of how the environment affects it and how the organism’s DNA affects it.

It is noteworthy that although cell concentration at time zero was not the same for all the treatments, the number of *E. coli* cells (Log CFU/gr) was analyzed compared to different storage (days) of each treatment in a row. Time 0 represents cell concentration after the saffron powder has been sprayed with E. coli. At that time, the samples underwent extensive analysis for the isolation and enumeration of the E. coli bacteria from other bacteria, considering the cultural characteristics of *E. coli* by the CFU method. We did this so all treatment groups could follow the same regimen, specifically the same storage duration times. Moreover, the initial cell concentrations of each treatment procedure did not affect the other one because the data analysis was conducted so that each group was compared to itself and only the changes between groups were discussed.

### Insufficient performance of cold plasma and packaging alone against saffron contamination

Furthermore, by evaluating CP and CNCs methods’ effects separately in *E. coli* inactivation, we showed that these modalities are not able to provide appropriate decontamination for saffron preservation. At doses that did not damage the quality of saffron, plasma alone was not able to eliminate microbial contamination. Only with irradiation for more than 10 min the total contamination disappears. However, in the latter case, the concentration of crocin, safranal, and picrocrocin is significantly reduced. Besides, the CNC packaging of saffron led to 0.9, 2, and 2.6 log CFU/gr reduction of *E. coli* after the 30, 60, and 90 day storage periods, respectively (Table 1). Therefore, although the CNCs packaging has shown good performance than CP alone, its performance during the storage period was poor compared to the CPs-CNC case.

These results go beyond the previous reports^32^, showing that only the synergy of CP and CNCs has high antibacterial activity and could eliminate all bacterial contaminations. Therefore, although adding nano clay gives antimicrobial properties to the CMC/PVA film, only the synergistic application of the CP and CNCs leads to a good barrier against microbial decontamination during the expected duration of saffron storage.

### Synergistic effect of nanocomposite packaging and plasma on saffron content

A further novel finding is a combination of CP and CPs-CNC that brings about highly interesting conditions for the safety and quality maintenance of saffron. Crocin and picrocrocin content was reduced by 6 and 0.30, respectively, in the A treatment group. Crocin and picrocrocin levels dropped by 2.9 and 0.6 in the group that got the D treatment, respectively. Moreover, for the D treatment group, 5.8 and 0.50 reductions in the content of crocin and picrocrocin have been observed, respectively. Meanwhile, safranal content has been increased for all treatment groups. Even though crocin, picrocrocin, and safranal concentrations changed after 90 days in conditions D and E, the alterations were small compared to A, B, and C.

As revealed in Table 2, after 90 days of storage, the amount of crocin, picrocrocin, and safranal of saffron packed in plastic bags were 12, 8.5, and 4.1%, respectively; whereas these amounts in the CP-CNC-D packed saffron were 14.1, 7.9, and 4%, respectively. Also, we have 10.40, 7.5, and 3.5% for crocin, picrocrocin, and safranal in the saffron samples packed in CP-CNC-E until 90 storage days. Moreover, the changes of picrocrocin and safranal in the control sample and the CPs-CNC packed saffron were approximately the same.

**Table 2:** The effect of the cold plasma treatment and the packaging on safranal, crocin and picrocrocin (%) of saffron.

Treatment	Package	Cold plasma treatment						
		Compounds	Voltage (kV)	Time (min)	Storage (Days)			
					0	30	60	90
A	Plastic bags	crocin	–	–	18.00 ± 0.17^a^	18.0 ± 0.22^a^	15.00 ± 0.32^b^	12.00 ± 0.66^c^
		picrocrocin			08.80 ± 0.24^a^	08.6 ± 0.63^b^	08.60 ± 0.42^b^	08.50 ± 0.21^c^
		safranal			03.20 ± 0.30^a^	03.4 ± 0.10^b^	03.70 ± 0.51^c^	04.10 ± 0.17^d^
B	CNC	crocin	–	–	18.00 ± 0.17^a^	18.00 ± 0.36^a^	16.0 ± 0.08^b^	15.00 ± 0.29^c^
		picrocrocin			08.80 ± 0.24^a^	08.80 ± 0.12^a^	08.60 ± 0.10^b^	08.40 ± 0.09^c^
		safranal			03.20 ± 0.30^a^	03.2 ± 0.46 ^a^	03.60 ± 0.08^b^	04.20 ± 0.11^c^
C	Plastic bags	crocin	7	10	08.00 ± 0.17^a^	07.90 ± 0.14^a^	05.40 ± 0.70^b^	04.00 ± 0.22^c^
		picrocrocin	7	10	05.70 ± 0.13^a^	05.50 ± 0.14^b^	05.30 ± 0.16^c^	05.00 ± 0.00^d^
		safranal	7	10	00.50 ± 0.00^a^	00.60 ± 0.11^b^	00.80 ± 0.03^c^	01.00 ± 0.62^c^
D	CNC	crocin	6	5	17.0 ± 0.41^a^	17.10 ± 0.35^a^	15.30 ± 0.12^b^	14.10 ± 0.71^c^
		picrocrocin	6	5	08.50 ± 0.27^a^	08.30 ± 0.00^b^	08.10 ± 0.36^c^	07.90 ± 0.40^d^
		safranal	6	5	03.00 ± 0.71^a^	03.10 ± 0.73^b^	03.60 ± 0.41^c^	04.00 ± 0.26^d^
E	CNC	crocin	7	3	16.20 ± 0.33^a^	16.10 ± 0.14^a^	11.30 ± 0.70^b^	10.40 ± 0.22^c^
		picrocrocin	7	3	08.00 ± 0.21^a^	07.90 ± 0.10^a^	07.80 ± 0.16^b^	07.50 ± 0.00^c^
		safranal	7	3	02.70 ± 0.19^a^	02.70 ± 0.11^a^	03.10 ± 0.03^b^	03.50 ± 0.62^c^

A: The Saffron is packed in a plastic bag without any treatment and stored for 30, 60, and 90 days. B: The Saffron is packed by PNC package without any treatment and stored for 30, 60, and 90 days. C: The Saffron is treated by cold plasma for 10 min at 7 kV (the time and voltage needed for complete decontamination) and then packed in a plastic bag without any treatment and stored for 30, 60, and 90 days. D: The CNC packed Saffron is treated by cold plasma for 5 min at 6 kV and stored for 30, 60, and 90 days. E: The CNC packed Saffron is treated by cold plasma for 3 min at 7 kV and stored for 30, 60, and 90 days. Safranal, crocin, and picrocrocin (%) were enumerated during treatment. abcd means in the same row followed by different superscript letters are significantly different (*P* < 0.05). Data represent the mean ± standard deviation of each sample (n = 3).

Although there is a lack of studies on the toxicity of interaction between the plasma generated reactive agents (RAs) (e.g., UV radiation, shock wave, ROS/RNS, EM field, etc.), and food compounds, high dose of RAs can cause harmful effects on food compounds. To avoid these undesirable effects, CP treatment should be used in low to medium doses, which is achievable by adjusting the plasma process parameters^81^. On the other hand, along with the high thermal resistance, cellulose-based films act as a shield from ultraviolet radiation and can carry bioactive materials^18^. Thus, CNCs can not only protect saffron components from plasma-generated UV radiation. Also, it can address the challenge of damaging saffron contents by controlling the release of plasma-generated ROS/RNS to saffron at desirable doses, which are not harmful to its contents.

### Insufficient performance of cold plasma and packaging alone on saffron content

Under conditions when the plasma alone has good antimicrobial properties, it reduces the concentrations of crocin, picrocrocin, and safranal. Reduction in crocin, picrocrocin and safranal content were 64 and 29 and 82% for the application of CP on saffron content after 12 min at 6 kV. The reduction of crocin, picrocrocin, and safranal content was 64 and 36, and 85% after 10 min of the CP treatment 7 kV (Table 3). Besides, our results show that the amount of safranal and picrocrocin is the same in all samples but the reduction of the crocin in CNC packed saffron is lower than in control (Table 2). From these results, it is clear that the combination of CP and CNCs shows much better performance than CP alone without affecting the saffron quality. Our group previously showed that saffron is sensitive to cold plasma treatment and the trans-4 GG, trans-3 Gg, trans-2 G, cis-4 GG, cis-3 Gg, isophorone, 4-Ketoisophorone, and safranal were changed, which is due to cleaving the Glycosidic linkages between monosaccharide in a polysaccharide chain of crocin and oxidative breakdown of safranal by free radicals^45^. The reduction of safranal after the cold plasma treatment may be due to the oxidative breakdown of safranal due to the formation of isophorone-related compounds^45^.

**Table 3:** The effect of the cold plasma treatment on safranal, crocin, and picrocrocin (%) of saffron.

	Voltage (kV)	Treatment time (min)					
		0	3	5	7	10	12
crocin	6	18.0 ± 0.17^a^	18.0 ± 12^a^	16.9 ± 0.4^b^	13.0 ± 1.4^c^	11.0 ± 0.7^d^	8.5 ± 0.10^e^
picrocrocin	6	08.8 ± 0.24^a^	8.7 ± 0.10^a^	8.6 ± 0.3^a^	8.3 ± 2.0^b^	7.00 ± 0.1^c^	6.3 ± 0.0^d^
safranal	6	3.2 ± 0.3^a^	3.2 ± 0.0^a^	3.0 ± 0.6^b^	0.2 ± 0.5^c^	1.1 ± 0.15^d^	0.6 ± 0.9^e^
crocin	7	18.0 ± 0.17^a^	16.1 ± 0.18^b^	12.5 ± 04^c^	9.9 ± 16^d^	0.8 ± 0.11^e^	–
picrocrocin	7	08.8 ± 0.24^a^	08.4 ± 0.10^b^	8.0 ± 0.31^c^	7.3 ± 0.25^d^	05.7 ± 0.14^e^	–
safranal	7	03.2 ± 0.30^a^	02.7 ± 0.10^b^	02.0 ± 0.45^c^	1.0 ± 0.19^d^	00.5 ± 0.00^e^	–

The Saffron is treated by cold plasma at different times (0, 3, 5, 7, 10, and 12 min) and different voltages (6 and 7 kV). The safranal, crocin, and picrocrocin were enumerated after treatment. abcdef means in the same row followed by different superscript letters are significantly different (*P* < 0.05). Data represent the mean ± standard deviation of each sample (n = 3).

### Role of device and process parameters of cold plasma

When comparing our results to those of the previous studies^45^, it must be pointed out that our findings can be used for plasma process optimization. As important process parameters, the voltage and exposure time in the CP treatment has significant effects on eliminating pollution and ensuring food safety. The data (Tables 3 and 4) verify that lower voltages and longer treatment time (5 min at 6 kV) resulted in better quality and more effective decontamination than the higher voltage and shorter treatment time (3 min at 7 kV). A comparison of the results of Table 3 shows that CNC packaging led to the smaller reduction of crocin during the storage which is because of the properties of the CNC package. As can be seen from this result, the selection of package materials and device and process parameters influencing the efficiency of CP are very important factors for each specific target in the food industry.

**Table 4:** The number of *E. coli* cells (Log CFU/gr) after the cold plasma treatment.

Voltage (kV)	Treatment time (min)					
	0	3	5	7	10	12
6	6 ± 0.45^a^	4.5 ± 0.82^b^	2.6 ± 0.9^c^	1.00 ± 3^d^	0.6 ± 20^e^	0.4 ± 18^f^
7	6 ± 0.45^a^	3.9 ± 0.16^b^	3.3 ± 12^c^	1.5 ± 0.5^d^	0^e^	0^f^

The Saffron is treated by cold plasma at different times (0, 3, 5, 7, 10, and 12 min) and at different voltages (6 and 7 kV). The number of *E. coli* was enumerated after treatment. abcdef means in the same row followed by different superscript letters are significantly different (*P* < 0.05). Data represent the mean ± standard deviation of each sample (n = 3).

This study aimed to scrutinize two basic questions in saffron preservation, especially during the present COVID-19 pandemic condition, through the synergy of CP and CNCs. Precise studies on the effect of CP treatment on CNCs and molecular mechanisms of *E. coli* inactivation are outside the scope of our work and can be implemented in future research.

## Conclusion

Herein, the high potential of the combined application of nanocomposite packaging and plasma treatment leads to food product safety, complete bacterial decontamination, and extended shelf-life in saffron preservation. The synergistic use of the plasma and nanocomposite packaging outperforms the current methods in saffron preservation when these and other methods are used separately. Microbial contamination studies demonstrated the effective combination of CNCs and CP for complete decontamination under conditions of low plasma exposure, where the quality of saffron is not affected. Importantly, our findings provide a mildly reactive environment for safranal, crocin, and picrocrocin during storage as the main content of saffron safety through the synergistic application of CP and CNCs. We have further investigated the factors influencing the performance of these modalities. Along with the selection of the packaging materials, device and process parameters such as the plasma exposure time and input voltage play a critical role in the efficacy of CP and CNCs modalities and must be carefully adjusted in future works. Future research on saffron decontamination procedure might extend the explanations of precise microbial inactivation mechanisms. Overall, we believe our findings contribute to the rapidly growing body of research aiming to find the best combination of advanced food processing and preservation technologies to overcome the major challenges in the preservation of saffron and a broad range of other valuable food products.

## Data Availability

All data generated or analysed during this study are included in this published article.
